# Supplementary material for: Association between oral microbiome and seven types of cancers in East Asian population: a two-sample Mendelian randomization analysis
Source: Front Mol Biosci. 2023 Nov 21;10:1327893. doi: 10.3389/fmolb.2023.1327893 (PMC10702768; doi:10.3389/fmolb.2023.1327893)
Supplement: Supplementary file 1 [file DataSheet1.ZIP › Supplementary Materials/MR plots of saliva/saliva═╝/Gastric cancer/pheno.3230_to_gastric cancer_forest.pdf]

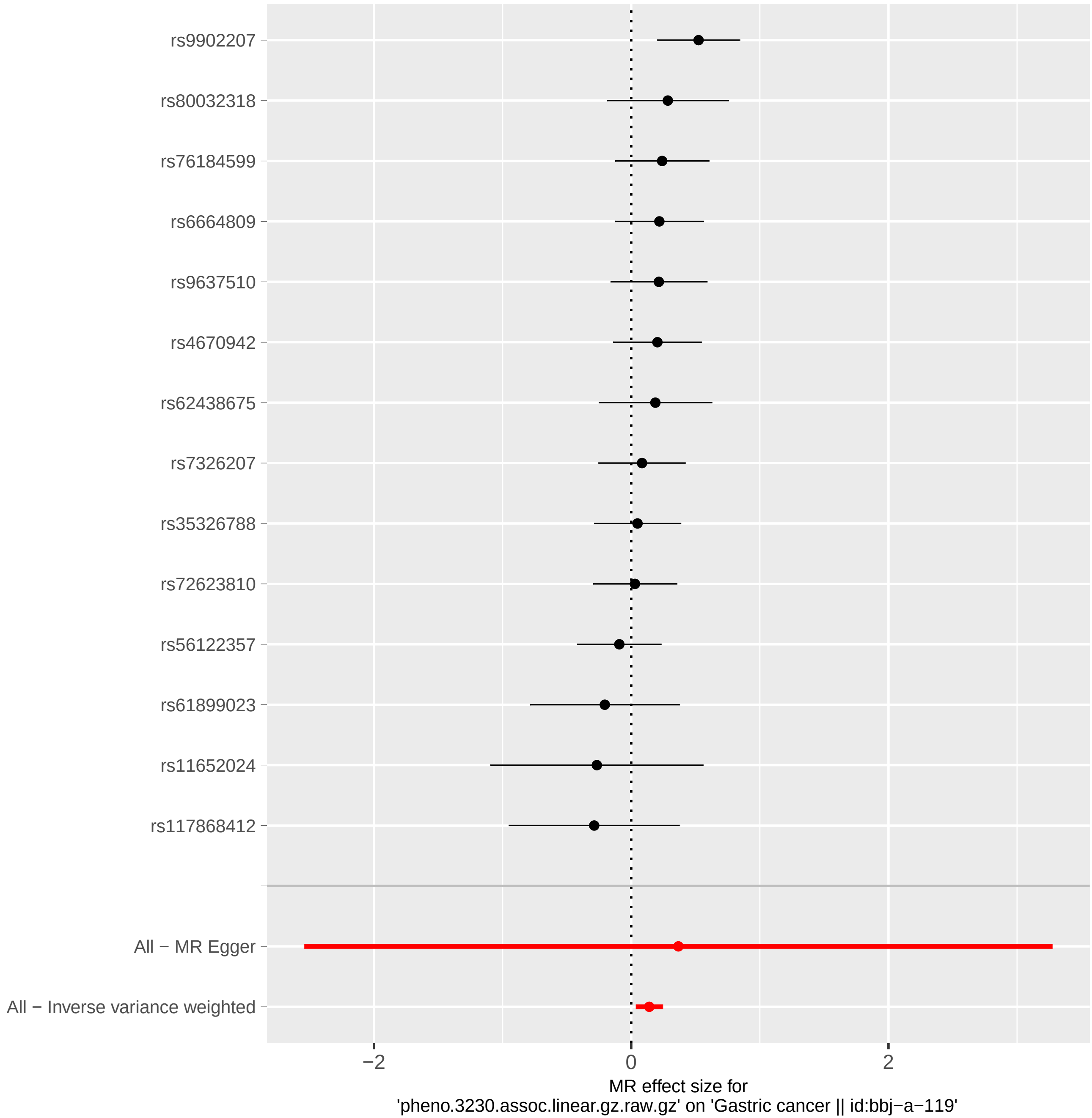

rs9902207
rs80032318
rs76184599
rs6664809
rs9637510
rs4670942
rs62438675
rs7326207
rs35326788
rs72623810
rs56122357
rs61899023
rs11652024
rs117868412
All − MR Egger
All − Inverse variance weighted
−2
0
2
MR effect size for
'pheno.3230.assoc.linear.gz.raw.gz' on 'Gastric cancer || id:bbj−a−119'
